# The Dichotomous Role of Bone Marrow Derived Cells in the Chemotherapy-Treated Tumor Microenvironment

**DOI:** 10.3390/jcm9123912

**Published:** 2020-12-02

**Authors:** Avital Vorontsova, Tal Kan, Ziv Raviv, Yuval Shaked

**Affiliations:** Rappaport-Technion-Integrated Cancer Center, Cell Biology and Cancer Science, Faculty of Medicine, Technion 3525422, Israel; avital.vor@gmail.com (A.V.); talkan11@gmail.com (T.K.); zraviv06@gmail.com (Z.R.)

**Keywords:** metastasis, chemotherapy, host response, immune cells

## Abstract

Bone marrow derived cells (BMDCs) play a wide variety of pro- and anti-tumorigenic roles in the tumor microenvironment (TME) and in the metastatic process. In response to chemotherapy, the anti-tumorigenic function of BMDCs can be enhanced due to chemotherapy-induced immunogenic cell death. However, in recent years, a growing body of evidence suggests that chemotherapy or other anti-cancer drugs can also facilitate a pro-tumorigenic function in BMDCs. This includes elevated angiogenesis, tumor cell proliferation and pro-tumorigenic immune modulation, ultimately contributing to therapy resistance. Such effects do not only contribute to the re-growth of primary tumors but can also support metastasis. Thus, the delicate balance of BMDC activities in the TME is violated following tumor perturbation, further requiring a better understanding of the complex crosstalk between tumor cells and BMDCs. In this review, we discuss the different types of BMDCs that reside in the TME and their activities in tumors following chemotherapy, with a major focus on their pro-tumorigenic role. We also cover aspects of rationally designed combination treatments that target or manipulate specific BMDC types to improve therapy outcomes.

## 1. Introduction

The tumor milieu is composed of both tumor cells and accessory cells. The accessory cells, which either reside in the tumor microenvironment (TME) or are recruited to it, support tumor growth and spread [1]. Immune cells, such as myeloid derived suppressor cells (MDSCs), macrophages, and specific immunosuppressive lymphocytes, regulate the activity of the adaptive immune system, thereby supporting pro-tumorigenic activities. For example, T regulatory cells (Tregs) promote tumor growth by homing to the TME where they modulate the immune system, allowing tumor cells to escape anti-tumor immune activity. In addition, macrophages recruited to the TME usually exhibit an immunosuppressive pro-tumorigenic state. They sustain tumor growth by inducing lymphangiogenesis and angiogenesis, and promote the spread of tumor cells from the primary tumor [2]. Non-immune cell types, usually originating from the mesoderm, infiltrate the TME and contribute to additional hallmarks of cancer. For example, endothelial cells support the sprouting of blood vessels into the tumor, leading to tumor oxygenation and subsequent growth [3]. Fibroblasts and pericytes enhance the rigidity of tumor blood vessels and induce the formation of tumor-promoting extracellular matrix (ECM) [4]. Recently, adipose cells were shown to promote low-grade inflammation in tumors, secreting adipokines and cytokines that support tumor growth [5]. Thus, the TME serves as a supportive bed for a growing community of various cell types, each of which participate in the growth of the tumor mass.

Chemotherapy remains one of the main treatment modalities for cancer. It acts by directly killing actively dividing cells, such as cancer cells. It is usually administered at the maximum tolerated dose (MTD) in cycles of one to three-week periods, followed by drug-free break periods [6]. These highly toxic concentrations of the drug are used with the intention of killing as many tumor cells as possible, whereas the drug-free break periods are necessary for decreasing side effects and host toxicities in order to promote healthy tissue recovery. These drug-holidays, however, allow for the recovery of tumor cells as well, and therefore can contribute to tumor cell repopulation and acquired resistance to therapy [6]. Therapy resistance has been studied for over a half century, with the majority of studies focusing on the mutations accumulating in the rapidly dividing tumor cells and the resulting tumor cell heterogeneity. Such effects lead to tumor clones that are less sensitive to the administered chemotherapy, explaining tumor cell proliferation despite continued drug use. Thus, a specific drug affects only some sub-populations of tumor cells within the tumor mass. The unaffected tumor cell populations, which are either intrinsically resistant to therapy or have developed resistance with time, repopulate the tumor mass [7].

In the last decade, an additional mechanism for acquired resistance to anti-cancer therapy has been proposed by our group followed by others. Specifically, we demonstrated that following chemotherapy, a host-mediated systemic effect is generated, which in turn supports tumor growth and spread. Several studies have shown that chemotherapy induces a rapid elevation of various chemokines, cytokines and growth factors followed by rapid mobilization of pro-tumorigenic cells that home to the treated TME leading to tumor re-growth. These host responses to therapy generate pro-tumorigenic and pro-metastatic biological pathways, such as angiogenesis, tumor cell epithelial-to-mesenchymal transmission (EMT) and cell proliferation, explaining, in part, acquired resistance to therapy [8]. For example, we demonstrated that upon the administration of certain chemotherapy drugs, there is an influx of circulating endothelial precursor cells (CEPs) which home to the treated TME and induce systemic angiogenesis leading to re-growth of tumors [9,10]. In another study, we showed that chemotherapy induces upregulation of MMP-9 specifically in BMDCs, an effect which facilitates EMT in tumor cells and supports their dissemination from the primary tumor site. Evidently, the deficiency of MMP-9 expression only in BMDCs reduced metastasis and increased survival in chemotherapy-treated mice [11]. These findings and others demonstrate that the host response to chemotherapy counteracts the anti-tumor activity of the drug, and therefore can explain tumor re-growth and acquired resistance to some extent.

**Figure 1:**
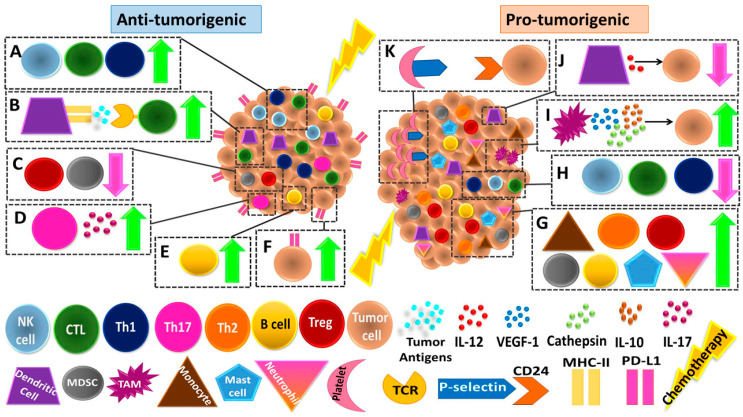
**Pro-tumorigenic and anti-tumorigenic roles of BMDCs in the chemotherapy-treated microenvironment.** The illustration provides examples describing the anti-tumorigenic (left panel, **A**–**F**) and pro-tumorigenic (right panel, **G**–**K**) host responses to chemotherapy, and their effects on the tumor microenvironment of different tumor types. Specifically, the anti-tumorigenic host effects consist of elevated levels of Th1, NK and CD8+ cytotoxic T cells (CTLs) in the TME (**A**); elevated tumor antigens presentation by dendritic cells to CTLs, further activating them (**B**); reduced infiltration of MDSCs and Tregs (**C**); increased IL-17 production by Th17 T cells (**D**); increased infiltration of B cells to the TME (**E**); and elevated expression of PDL-1 by tumor cells (**F**). On the other hand, the pro-tumorigenic host effects involve immune host cells including monocytes, T helper 2 (Th2) cells, T regulatory cells (Tregs), myeloid derived suppressor cells (MDSCs), B cells, mast cells and neutrophils, which are upregulated in the TME (**G**); immune host cells including T helper 1 (Th1), natural killer (NK) cells, and CD8+ cytotoxic T cells are downregulated in the TME (**H**); tumor-associated macrophages (TAMs)secreting IL-10, cathepsins and VEGF-C are infiltrating the treated TME further supporting tumor re-growth and metastasis (**I**); IL-12 production levels are downregulated in dendritic cells following chemotherapy treatment, resulting in decreased granzyme B production by CTLs, further contributing to tumor growth and metastasis (**J**); and activated platelets aggregate in the TME, further binding to tumor cells via P-selectin-CD24 axis (**K**).

The balance between anti- and pro-tumorigenic activities generated in response to chemotherapy raises questions regarding the activity of different cell types within the TME. As opposed to the notion that chemotherapy specifically targets and affects primarily tumor, it is currently clear that the effects are more general and probably extensive, as it also affects non-tumor (host) cells. Studying the effect of chemotherapy on host cell types within the TME can provide further insights into tumor-host communications. The host responses to chemotherapy may vary depending on tumor type and TME composition. For example, it is known that the TME of sarcomas differs to that of carcinomas (reviewed in [12]). In this review, we focus mainly on the effects of chemotherapy on host and tumor cells with special emphasis on carcinoma tumor types. Understanding the complexity of the pro- and anti-tumorigenic activities, as illustrated in Figure 1, can shed light on new ways to treat cancer and improve the efficacy of commonly used chemotherapy drugs. In this review, we will discuss the role of different BMDCs in the TME, with a focus on their various and sometimes conflicting roles in response to chemotherapy.

## 2. Discussion

### 2.1. The Anti-Tumorigenic Effect of Immune Cells Following Chemotherapy

#### 2.1.1. The Induction of Immunogenic Cell Death

To elicit an effective immune response against tumor cells, tumor antigens must be presented by antigen presenting cells (APCs) to CD8+ T cells (CTLs). The antigen specific CD8+ T cells migrate into the tumor and support anti-tumorigenic functions. However, many immunosuppressive mechanisms are hijacked by the tumors to inhibit the cytotoxic activity of T cells, and also to inhibit their recruitment to the tumor and promote their exhaustion [13] (Figure 1A). While this is a common situation in spontaneously growing tumors, the immune process is different following chemotherapy. Specifically, chemotherapeutic agents are mainly considered to target and kill malignant cells, but at the same time, they also affect immune cells and alter their function. Indeed, various chemotherapeutic agents have been shown to promote immunogenic cell death (ICD) [14]. By doing so, they boost an anti-tumor immune response, which is largely mediated via dendritic cells (DCs) (Figure 1B). DCs are key initiators of a T-cell-specific response and the most potent APCs. They are a heterogeneous cell population which exert mostly anti-tumorigenic roles. The levels of DCs inversely correlate with tumor progression. DC subsets were shown to react differently in chemotherapy-induced ICD [15]. For example, conventional type 1 DCs (cDC1) effectively present tumor antigens to CD8+ T cells and elicit an anti-tumor immune response [16]. ICD induced by mitoxantrone was found to promote ATP release from dying cells which favored cDC1-like cell differentiation in the TME. In turn, these cDC1 cells present tumor antigens to CD8+ cells and promote anti-tumor immunity [17]. However, DC function in response to chemotherapy is not limited to ICD only. It has been shown that following paclitaxel, doxorubicin and vinblastine chemotherapies, bone-marrow derived DCs (BM-DCs) express stimulatory molecules including IL-12, CD80, CD86 and major histocompatibility complex (MHC) II, which in turn induce the activation of CD8+ T cells [18,19]. Thus, the levels of DCs in peripheral blood and the TME may be used as a predictive biomarker for anti-tumor immunity following chemotherapy. It should be noted, however, that chemotherapy, on its own, can directly affect the viability of DCs, and thus can compromise their activity. Nevertheless, DCs are resistant to some chemotherapies such as etoposide, 5-FU and paclitaxel [20,21], indicating that the direct response of DCs to specific chemotherapies may determine prognosis in patients. Overall, these results demonstrate the anti-tumorigenic role of DCs in the immune response against tumors and its association with chemotherapy-induced ICD.

#### 2.1.2. The Anti-Tumorigenic Role of Lymphoid Cells

Anti-tumor immunity is also associated with the suppression of various subsets of CD4+ regulatory T cells (Figure 1C). In a preclinical model of mice bearing lung carcinoma, paclitaxel has been demonstrated to selectively inhibit CD4+FOXP3+ Treg cells, but not other CD4+FOXP3- T helper cells [22]. The inhibition of Tregs supports CD8+ T cell activity [23]. Clinically, peripheral blood obtained from non-small cell lung cancer (NSCLC) patients at baseline displayed high levels of immunosuppressive Treg and T helper type 2 (Th2) cell populations while the anti-tumorigenic T helper type 1 (Th1) cells were low. However, after two cycles of chemotherapy, including cisplatin/pemetrexed or nedaplatin/pemetrexed, the levels of Treg and Th2 cells decreased and the levels of Th1 cell population significantly increased. This study indicated that high peripheral blood levels of Th1 cells and low levels of Treg cells were significantly correlated with progression-free survival (PFS) [24]. Moreover, in the TME of most human breast cancers, T helper cells mostly present a Th2 phenotype. However, following treatment with chemotherapy, T helper cells are shifted from the pro-tumorigenic Th2 to the anti-tumorigenic Th1 phenotype, resulting in a higher level of CD8+ T cells [25] (Figure 1A). The changes in Th cells following chemotherapy may explain increased tumor infiltrating lymphocytes (TILs) in the TME of triple negative and HER2 positive breast carcinoma patients treated with carboplatin [26], thus demonstrating the effective role of chemotherapy in skewing a Th2 to Th1 phenotype within the tumor and therefore contributing to anti-tumor TILs. 

Another CD4+ T cell subset which plays a role in tumor immunity is the Th17 cell subset. These cells can exert both anti- and pro-inflammatory responses [27]. Therefore, their contribution to cancer progression or inhibition is somewhat controversial [28]. However, studies reported that following chemotherapy, Th17 cells produce high levels of IL-17, which in turn contributes to anti-tumor immunity [29] (Figure 1D). For example, in ovarian tumor biopsies, high IL-17 levels were associated with response to platinum-based chemotherapies [30]. These effects were also reported for patients with melanoma and ovarian cancer treated with cyclophosphamide [31]. While the mechanisms involved in chemotherapy-induced Th17 anti-tumor activity are not clear, a recent study suggested that it is associated with changes of microbial content in the gut in response to chemotherapy. These changes stimulate a specific pathogenic Th17 and Th1 immune response that likely promotes anti-tumor activity [32]. Overall, the modulation of specific T cell subsets within the TME in response to chemotherapy contributes to increased anti-tumor cytotoxic T cell activity.

Recent studies have also demonstrated the significance of B cell anti-tumor activity in response to chemotherapies (Figure 1E). In mice, inducible T cell costimulatory ligand (ICOSL)+ B cell population was dramatically increased in response to chemotherapy and was dependent on complement receptor type 2 (CR-2) activation. ICOSL+ B cells were found to elicit an improved anti-tumor immune response following chemotherapy, which was reverted by depleting B cells with an antibody or in a transgenic mouse model lacking mature B cells [33]. Clinically, tumor biopsies from triple negative breast cancer patients treated with neoadjuvant chemotherapy displayed a substantial increase in the frequency of ICOSL+ B cells in comparison to their biopsies before the treatment. This specific B cell population was highly associated with better prognosis [33]. In addition, B cells have also been proposed to contribute to a better prognosis in muscle-invasive bladder cancer patients receiving adjuvant platinum-based chemotherapy. Specifically, high frequency of stromal tumor infiltrating B cells (TIBs) correlated with improved overall survival (OS) in patients treated with adjuvant chemotherapy, while those with low TIBs did not display any correlation [34]. These B cells were located in the treated tumors in close proximity to CD4+ T cells and had high MHCII expression, serving as an explanation for their overall anti-tumor activity [34]. According to the TCGA gene expression data analysis, enrichment of antigen presentation signaling, and T cell-mediated immunity was found in patients with high levels of TIBs [34]. Thus, like T cells, B cells also support anti-tumor immunity. 

Chemotherapy may change the function of NK cells by elevating the expression of natural cytotoxicity receptors (NCRs) and other NK-related receptors. For example, in metastatic melanoma patients, NKp46 and NKG2A expression were elevated in NK cells following chemotherapy treatment, further inducing cytotoxic activity towards melanoma cells [35]. In another study, it has been suggested that the response of cancer cells to various chemotherapeutic agents can augment cytokine secretion, leading to the activation of NK cells. Specifically, melphalan, a chemotherapy used to treat multiple myeloma (MM) patients, promotes exosome release from MM cells which in turn induces IFN-γ secretion from NK cells [36]. In addition, bortezomib was found to upregulate the expression of NK cell ligands on cancer stem cells (CSCs), leading to enhanced NK cell anti-tumor effects [37]. Taken together, the aforementioned studies further extend our understanding of the anti-tumor immune effects associated with the suppression of immune regulatory cells and the activation of tumor-specific immune cells in response to chemotherapy.

#### 2.1.3. The Modulation of Immune-Checkpoint Molecules

Chemotherapy has been shown to promote anti-tumor immunity by upregulating the expression of immune checkpoint molecules in tumors. Specifically, PD-L1 expression was shown to be upregulated after neoadjuvant chemotherapy in urothelial carcinoma [38] and in NSCLC [39,40] as well as in adjuvant settings in NSCLC [41] (Figure 1F). Increased PD-L1 expression in tumor cells and their extracellular vehicles (EVs) were also found after tumor cells were exposed to radiotherapy [42]. These findings support the rationale for combining chemotherapy and immune checkpoint inhibitors (ICIs), since the upregulation of PD-L1 in tumor cells sensitizes them to immune checkpoint therapy. Consistently, in preclinical models, it was shown that treating lung adenocarcinoma bearing mice with oxaliplatin/cyclophosphamide combination promoted T cell influx into the tumor, sensitizing it to checkpoint blockade therapy [43]. Indeed, clinically, the combination of atezolizumab (anti-PD-L1) with platinum-based chemotherapy for metastatic non-squamous NSCLC was approved as a first line therapy based on a recent phase III study [44]. Thus, the immune modulation of T cells is also dependent on the expression of checkpoint molecules by tumor cells exposed to chemotherapy.

### 2.2. The Pro-Tumorigenic Effects of Immune Cells Following Chemotherapy

#### 2.2.1. Pro-Tumorigenic Lymphoid Cells

Chemotherapy is known to eliminate immune cells and promote myelosuppression. Therefore, it can alter the balance between myeloid and lymphoid cells, leading to tumor growth and spread (Figure 1G) [45]. In this regard, clinical outcomes in patients with invasive urothelial carcinoma of the bladder treated with cisplatin-based neoadjuvant chemotherapy were dependent on the ratio of CD8+ to Treg TILs. A ratio lower than 1 predicted a worse outcome following treatment [46], indicating that chemotherapy disrupts this ratio and thus contributes to tumor re-growth. As infiltrating T helper cells, specifically IFN-γ producing CD4+ Th1 cells, usually possess an anti-tumorigenic role in the TME [47], their elimination in the treated tumor due to chemotherapy supports pro-tumorigenic activity. Indeed, a reduced number of Th1 cells has been shown to correlate with poor prognosis in response to chemotherapy [24,48]. Likewise, breast cancer patients treated with adjuvant chemotherapy displayed an elevated number of circulating tumor cells (CTCs) that correlated with a reduced number of total T helper cells in the peripheral blood, further suggesting chemotherapy-induced inhibition of active T helper cells [49]. Overall, levels of T helper cells in the TME and peripheral blood are reduced following chemotherapy, ultimately facilitating tumor cell growth and spread.

In addition to TILs, B cells may also exert pro-tumorigenic activity (Figure 1G). For instance, metastatic ovarian carcinoma patients who at baseline displayed high levels of TIBs and were subsequently treated with platinum-based chemotherapy, exhibited a decreased OS [50]. These pro-tumorigenic activities of B cells could be due to CD19+ EVs originating from B cells and impaired anti-tumorigenic activities of CD8+ T cells. It has been shown that ATP is hydrolyzed into adenosine by tumor cells in response to chemotherapy. Adenosine induces immunosuppressive activities, and therefore promotes tumor growth, invasion and metastasis [51]. Regulatory B cells (Bregs) are another cell type found to promote immunosuppressive activity due to the secretion of anti-inflammatory cytokines, such as IL-10 and the production of adenosine [52]. In head and neck squamous cell carcinoma (HNSCC) patients, methotrexate therapy increased Bregs and further promoted anti-inflammatory adenosine production, thereby supporting immunosuppressive activity within the treated TME [53]. Thus, B cells may possess immunosuppressive activities within the chemotherapy-treated TME, especially if they produce adenosine, which in turn inhibits the cytotoxic effect of CD8+ cells [54].

The anti-tumorigenic role of NK cells is already well covered [55], therefore, their elimination by chemotherapy may lead to pro-tumorigenic activity (Figure 1H). For example, NK cells are drastically reduced following anthracyclines and cytarabine chemotherapy treatment in elderly patients with AML [56]. In addition, the systemic elimination of CD16+ NK cell populations in colorectal cancer patients treated with FOLFOX (5-floroucil, oxaliplatin and leucovorin) chemotherapy decreased anti-tumorigenic cytokine production. Thus, chemotherapy likely eliminates NK cell anti-tumor activity, and therefore contributes to the pro-tumorigenic process. Overall, these findings demonstrate that chemotherapy may modulate the immune system in favor of the tumor, via increased activity of immune-regulatory cells and decreased viability of anti-tumor immune cells.

#### 2.2.2. Pro-Tumorigenic Myeloid Cells

##### Tumor-Associated Macrophages

Myeloid cells are known to support tumor growth and spread, an effect which is enhanced in response to chemotherapy, as recently reviewed [57]. In the TME, macrophages are usually in an M2-like immunosuppressive state and they are mainly referred to as tumor associated macrophages (TAMs) (Figure 1I). TAMs support tumor growth by various biological pathways including angiogenesis and directly contribute to tumor cell proliferation [57]. However, after chemotherapy, macrophages become more potent in their ability to induce biological pathways that support tumor re-growth and spread. For example, in lung and breast carcinoma models, TAMs secrete cathepsins and VEGF-C following paclitaxel chemotherapy. This in turn supports lymphangiogenesis and ultimately metastasis [58,59]. In addition, in breast carcinoma patients treated with doxorubicin and cyclophosphamide, TAMs secrete cathepsins that protect tumor cells from the cytotoxic effect of the chemotherapy drugs [59]. Similarly, cathepsin Z was shown to protect tumor cells from chemotherapy in pancreatic neuroendocrine tumor xenografts [60]. Thus, in response to chemotherapy, TAMs secrete ECM-related enzymes which protect tumor cells from the cytotoxic effects of the drug.

The contribution of TAMs to tumor growth and metastasis was recently described in murine xenograft models of breast cancer. A specific phenotype of TIE2hi/VEGFhi macrophages as well as other BMDCs that infiltrate the chemotherapy-treated TME, especially following neoadjuvant paclitaxel after doxorubicin and cyclophosphamide therapy, contributed to the dissemination of tumor cells from the primary tumor site in a unique process [61]. The mechanism involves high expression of MENAINV isoform by tumor cells [62], which in turn supports the formation of activated sites of tumor microenvironment of metastasis (TMEM). These TMEMs open the doorway for tumor cells to escape the primary tumor site. Therefore, blocking the activity of TAMs in response to chemotherapy may improve clinical outcomes. Indeed, depleting macrophages using CSF1-R-signaling antagonists improved outcomes in human breast cancer xenografts following combination chemotherapy (cyclophosphamide, methotrexate, and 5-fluorouracil) [63] and in murine PyMT models following paclitaxel treatment [64].

##### Monocytes and Myeloid-Derived Suppressor Cells

MDSCs and monocytes support tumor growth in response to chemotherapy by inducing angiogenesis, immunosuppressive activities or directly contributing to tumor resistance (Figure 1G). In response to chemotherapy, these cells secrete a variety of factors associated with pro-tumorigenic activity. For example, monocytic MDSCs were shown to contribute to lymphoma resistance following doxorubicin therapy by reducing caspase-3 activity and increasing heat shock protein-27 (Hsp27) signaling in tumor cells. Hsp27 signaling protected tumor cells from apoptosis [65]. Likewise, MDSCs secrete IL-1β due to elevated activation of the inflammasome complex by gemcitabine or 5-FU chemotherapies. The release of IL-1β decreased anti-tumor immune activity, thereby supporting tumor resistance to chemotherapy in murine lymphoma, melanoma and Lewis lung carcinoma [66]. Despite these findings, it has been demonstrated that the blockade of chemotherapy-induced IL-1β by IL-1 receptor antagonist resulted in increased angiogenesis and metastasis [67]. Thus, while IL-1β-induced MDSCs in response to chemotherapy support pro-tumorigenic activity, it is possible that the effects are mostly associated with resistance to chemotherapy at the primary tumor site, where the blockade of IL-1β following chemotherapy may increase pro-metastatic biological processes. Indeed, it has been documented that chemotherapy pushes the phenotype of monocytes into M2, immunosuppressive macrophages, thereby supporting human cervical and ovarian cancer cell growth and spread [68]. Similarly, Tie-2 expressing monocytes were shown to home to the treated TME following cytotoxic-like agents, such as combretastatin, and contribute to tumor re-growth by inducing angiogenesis [69]. Taken together, monocytes support tumor growth by various pro-tumorigenic activities including angiogenesis, tumor cell proliferation and immunosuppressive activities within the TME.

##### Other Myeloid Cells

Mast cells, like other immune cells secrete various molecules such as cytokines, chemokines, and growth factors, known to modulate immunity sometimes to the benefit of tumors [70] (Figure 1G). Abundance of tumor infiltrating mast cells was shown to predict tumor progression in muscle-invasive bladder cancer (MIBC) following cisplatin adjuvant chemotherapy and in pancreatic cancer following gemcitabine/nab-paclitaxel therapy [71,72]. In contrast, high infiltration of mast cells was predictive of prolonged OS in biliary tract cancer patients following gemcitabine-based adjuvant chemotherapy and in gastric cancer following 5-FU adjuvant chemotherapy [73,74]. Another study reported that high levels of mast cells in the TME of inflammatory breast carcinoma following neoadjuvant chemotherapy correlated with poor response [75]. In prostate cancer, tumor infiltrating mast cells induce the expression of p21 in tumor cells, which activates the p38/p53 signaling. The activation of this signaling pathway promoted chemo-resistance in both in vitro and in vivo models [76]. Thus, the infiltration of mast cells into the chemotherapy-treated TME mostly supports chemo-resistance and poor response.

DCs, like other immune cells, exert mostly anti-tumorigenic activities as discussed above. However, a limited number of studies raised the possibility that DCs can sometimes promote tumor growth, usually via their interactions with other immune cells (Figure 1J). For example, while cDC1 elicit an anti-tumor immune response [16], its interactions with TAMs in the chemotherapy-treated TME contribute to pro-tumorigenic effects. Specifically, in murine breast carcinoma, TAMs secrete increased levels of IL-10 upon paclitaxel or carboplatin chemotherapy. As a result, cDC1 residing in the treated-TME express decreased levels of IL-12, leading to suppression of CD8+ T cells, and further supporting tumor growth [77]. Moreover, TIM-3 receptor expressed by DCs was shown to inhibit ICD of MC38 tumors following cisplatin chemotherapy. The mechanism involved the binding of TIM-3 to high mobility box 1 protein (HMGB1). This binding inhibits the internalization of nucleic acids derived from apoptotic tumor cells into DCs, which in turn suppresses their anti-tumor activity [78]. Overall, these findings suggest that chemotherapy contributes to a variety of pro-tumorigenic activities within the TME or in the peripheral blood, leading to tumor re-growth and spread.

#### 2.2.3. The Role of Platelets in the Chemotherapy-Treated TME

Tumor necrosis due to chemotherapy results in activation and aggregation of platelets in the TME, as part of the wound healing process (Figure 1K) [79]. The aggregation of platelets is a prognostic marker for worse outcome [80]. Specifically, a high platelet-to-lymphocyte ratio (PLR) in peripheral blood was associated with lower PFS, lower OS and poor response to chemotherapy [81]. The use of PLR as a marker of response and worse outcome has been evaluated for a number of indications, including colorectal cancer patients with liver metastasis [82], metastatic gastric cancer [83], metastatic triple negative cancer [84] and non-metastatic esophageal squamous cell carcinoma [85]. While the mechanism behind PLR is not covered, a study suggested that the elevation in the percentage of platelets in peripheral blood is indicative of systemic inflammation. Specifically, this inflammation involves an increased activity of neutrophils and lymphocytes which secrete factors associated with tumor progression, such as C-reactive protein and albumin [86]. Although the mechanisms by which C-reactive protein and albumin support tumor growth are not well known, the high ratio of these proteins in peripheral blood was shown to have a negative prognostic value in small cell lung carcinoma [87]. In addition, when platelets are aggregated in the TME, they become highly activated due to their binding to tumor cells via P-selectin-CD24 axis (Figure 1K). This activation leads to thrombosis and inflammation, processes that can explain poor prognosis [88]. Thus, high platelet levels in the peripheral blood are a potential indicator of worse prognosis in cancer patients, an effect which is augmented following chemotherapy due to tissue damage [89].

#### 2.2.4. BMDCs in Metastatic Sites in Response to Chemotherapy

Cancer cells tend to metastasize into selective secondary organs via various mechanisms. This concept of organotropism—organ selectivity to metastasize—was first introduced by Stephen Paget in 1889 as the “the seed and soil hypothesis” [90]. In recent years, it has been established that the specificity of metastasis to specific organs is dependent on the formation of a pre-metastatic niche [91]. The colonization of specific VEGFR-1+VLA-4+ hematopoietic progenitor cells (HPCs) in clusters contributes to the formation of an organ specific pre-metastatic niche. Subsequently, tumor cells seed in complexes of cells and support fibronectin production to form metastasis. A recent study indicated that these effects are probably enhanced in response to chemotherapies, such as cisplatin and paclitaxel. Specifically, elevated levels of VEGFR-1+ expressing endothelial cells in the pre-metastatic niche induces metastasis formation [92]. Whether these endothelial cells are similar to CEPs (with respect to expression of hematopoietic progenitor cell markers) has not been demonstrated. Nevertheless, it seems that chemotherapy supports metastasis by facilitating specific niche formation for tumor cells to seed. Likewise, another study demonstrated that cisplatin and vincristine chemotherapies can enhance the formation of liver pre-metastatic niches in B16F10 murine melanoma or BE(2)-C human neuroblastoma mouse models. These effects are mediated by the induction of MMP-2, periostin, MMP-9 and S100A8/9 levels in liver tissue [93]. It should be noted that increased MMP-2 and MMP-9, sometimes expressed by BMDCs, support epithelial-to-mesenchymal transition of tumor cells, allowing them to metastasize [11,94]. Evidently, blocking the expression of MMP-9 solely in BMDCs following paclitaxel chemotherapy resulted in reduced mortality rate, suggesting that MMP-9 expressed by BMDCs is critical for chemotherapy-induced metastasis [11].

EVs may also play a role in the formation of the pre-metastatic niche. For example, it was demonstrated in breast cancer mouse models that neoadjuvant chemotherapies such as taxanes and anthracyclines promote secretion of EVs from tumor cells, further supporting lung pre-metastatic niche formation. These effects are partially mediated by Ly6C+CCR2+ monocytes that are recruited to the pre-metastatic site and support the seeding of tumor cells [95]. Overall, chemotherapy can alter the formation of pre-metastatic sites by the secretion of systemic pro-metastatic factors, and thus promotes a favorable “soil” for metastasis formation.

### 2.3. The Role of Non-Hematopoietic Cells in Response to Chemotherapy

Non-hematopoietic cells were found to play a critical role in regulating and supporting tumor growth, similar to BMDCs. Specifically, mesenchymal stem cells (MSCs), cancer-associated fibroblasts (CAFs), endothelial cells and their precursor subset as well as adipocytes have been studied in the context of cancer [96]. A growing body of evidence indicates that such cells can generate pro-tumorigenic activities in response to chemotherapy. For example, upon chemotherapy, CAFs and MSCs support tumor growth by enriching CSCs, which are most likely resistant to many different anti-cancer drugs and can initiate tumorigenesis [97]. For example, the phenotype and function of MSCs was shown to be affected by chemotherapy when MSC pretreatment with cisplatin led to changes in phosphorylation profiles of protein kinases and increased secretion of IL-6 and IL-8 cytokines. These changes led to increased chemo-resistance and stemness of breast cancer cells [98]. In a murine pancreatic cancer model, following gemcitabine chemotherapy, MSCs were shown to colonize the TME in large numbers and reside in close proximity to CSCs, therefore suggesting their contribution to CSC niche. They were found to secrete CXCL10, which in turn, supports CSC enrichment, thereby contributing to tumor relapse and resistance [99]. Like MSCs, CAFs were also shown to support CSC enrichment. Specifically, treatment of pancreatic cancer with doxorubicin, paclitaxel or 4-hydroxy-cyclophosphamide (4H-CPA) chemotherapies resulted in CAF-induced STAT-1 and NF-κB activity leading to the secretion of ELR motif-positive (ELR+) chemokines. In turn, these ELR+ chemokines promote CSC enrichment through CXCR-2 signaling on tumor cells [100]. In line with these results, another study reported that a specific subset of CAFs, expressing CD10 and GPR77, was correlated with resistance to chemotherapy and poor survival of breast and lung cancer patients. These cells promoted tumor growth and resistance to chemotherapy by providing a survival niche for CSCs via NF-κB signaling [101]. Overall, both MSCs and CAFs support CSC enrichment in response to chemotherapy, and therefore can explain tumor re-growth and resistance. However, resistance is not only related to CSC enrichment. Recent studies have demonstrated that MSCs secrete specific polyunsaturated fatty acids that indirectly protect tumor cells from the cytotoxic effects of chemotherapy. These findings were demonstrated in vivo in colon carcinoma and Lewis lung carcinoma (LLC) mouse models, as well as human adenocarcinoma, treated with cisplatin, oxaliplatin, carboplatin and irinotecan chemotherapies [102]. In addition, MSCs were shown to protect ovarian cancer cells from cell death induced by hyperthermic intraperitoneal chemotherapy via the activation of CXCL12-CXCR4 axis. Blocking the CXCR4 axis restored the thermo-sensitivity of ovarian cancer cells [103]. The secretion of a variety of cytokines by MSCs following chemotherapy promotes their pro-tumorigenic activity. For example, IL-6 secreted by MSCs in response to paclitaxel or doxorubicin, resulted in the activation of STAT3 signaling pathway, promoting the growth of head and neck cancer, nasopharyngeal carcinomas as well as osteosarcoma [104,105,106]. Overall, MSCs and CAFs play a significant role in tumor resistance, in part by activating survival signaling pathways in cancer cells and enriching for CSCs that are known to resist different anti-cancer drugs.

Like MSCs and CAFs, tumor-associated adipocytes are involved in chemo-resistance [107]. For example, adipocytes were suggested to contribute to doxorubicin resistance in various breast cancer cell lines by promoting the expulsion of cytoplasmic vesicles containing doxorubicin from the treated cancer cells. This activity reduces the concentration of the drug in tumor cells and thus contributes to therapy resistance [108]. Likewise, adipocyte-derived conditioned medium or cells were found to protect breast, pancreatic, and melanoma cancer cells from various chemotherapy drugs such as gemcitabine, cisplatin and docetaxel chemotherapies [109,110,111]. In ovarian cancer, for example, adipocytes were shown to mediate chemo-resistance by secreting lipids, such as arachidonic acid. These lipids activate the Akt pathway in ovarian cancer cells exposed to cisplatin chemotherapy further protecting them from apoptosis [112]. Another example of adipocyte-mediated chemo-resistance has been demonstrated in relation to the adipokine leptin. Leptin increases chemo-resistance to 5-FU in pancreatic and colorectal cancers, resulting in increased tumor cell proliferation and reduced levels of pro-apoptotic factors, such as Bax, Caspase-3 and PARP [113,114]. A study revealed that bone marrow adipocytes protect MM cells from chemotherapy-induced cytotoxicity. They do so by secreting leptin and adipsin, which in turn upregulate autophagy proteins in MM cells. These effects lead to the suppression of caspase activity and reduced apoptosis [115]. 

Non-hematopoietic cells do not only support therapy resistance but can also contribute to tumor-re-growth by inducing angiogenesis. Our previous studies demonstrated that CEPs contribute to a rapid induction in angiogenesis, an effect which is most potent following chemotherapy [10]. We demonstrated that upon paclitaxel chemotherapy, CEPs infiltrate LLC tumors and induce the formation of new blood vessels [9]. The mechanisms of their homing to the treated TME is mediated in part by systemic expression of SDF-1α, a factor known to mobilize BMDCs, and thus support angiogenesis. These preclinical studies were also supported by clinical data demonstrating that circulating endothelial cells and CEPs can serve as surrogate markers for PFS and OS in patients with various cancer types, such as breast, colorectal, ovarian, esophagus and prostate, who were treated with a number of chemotherapy drugs [116,117]. Thus, while chemotherapy may partially kill dividing endothelial cells in growing tumors, upon therapy, such drugs rapidly mobilize CEPs and other bone marrow derived pro-angiogenic cells into the treated tumor site and support tumor growth by inducing angiogenesis [118,119].

### 2.4. Clinical Implications

**Figure 2:**
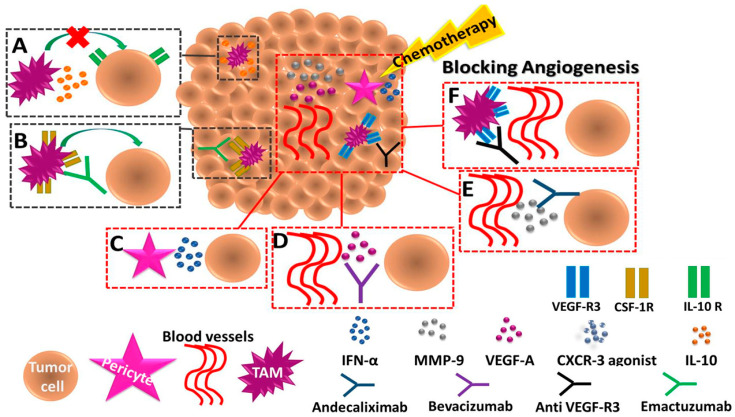
**Blunting pro-tumorigenic host responses to chemotherapy by treatment combinations.** The illustration provides examples describing ways to block host responses to chemotherapy in order to improve therapeutic outcome. The pro-tumorigenic activity of tumor associated macrophages (TAMs) found following chemotherapy can be targeted based on their function. Specifically, neutralizing IL-10 inhibits the immunosuppressive activity of TAMs, therefore supporting anti-tumor immunity (**A**); and blocking CSF1-R inhibits infiltration of pro-tumorigenic TAMs to the treated TME (**B**). In addition, an induction of angiogenesis following chemotherapy can be blocked by IFN-α which inhibits pericyte function (**C**); by neutralizing VEGF-A (**D**); and by blocking MMP-9 (**E**). Moreover, blocking VEGF-R3 inhibits TAM-induced lymphangiogenesis (**F**). These combined therapies can improve the therapeutic outcome of chemotherapy and inhibit metastasis.

Given that chemotherapies exert both pro- and anti-tumorigenic host-mediated activities, it is challenging to identify the patients who will benefit from a given therapy. In this regard, the analysis of host systemic and local response following chemotherapy and the identification of pro-tumorigenic biological pathways may help to predict clinical outcome of patients. We must note that some of the studies described above were performed when the chemotherapy drug was given only once and not constitutively or in cycles every 1–3 weeks. In addition, other studies have demonstrated that the host effects were separated from the anti-tumor effect by treating non-tumor bearing mice with chemotherapy. Yet, these results may explain why sometimes, even though patients display initial response to therapy, eventually they cease to respond and tumor re-growth or spread follows. Uncovering the mechanisms underlying the pro-tumorigenic host responses to chemotherapy creates a new therapeutic opportunity whereby host pro-tumorigenic responses are blocked with targeted drugs. Some of these strategies are described below and illustrated in Figure 2.

#### 2.4.1. Blunting Host Response with Treatment Combinations

The identification of molecular and cellular factors accountable for the different host responses to therapy may reveal new drug targets. Specifically, drugs that block or inhibit the key factors that promote tumor cell aggressiveness can be used in combination with the conventional chemotherapy treatment potentially improving therapy outcome. This strategy was demonstrated in several preclinical studies, when the blockade of pro-tumorigenic cells or factors are combined with chemotherapy. For example, IL-10 secreted by macrophages in response to paclitaxel or carboplatin chemotherapies was inhibited by therapeutically blocking the IL-10-IL-10R axis (Figure 2A). This combination therapy enhanced primary tumor response to chemotherapy and improved outcome of murine model of breast cancer. The improved response was mediated in part by a CD8+ T cell-dependent effect increasing intra-tumoral dendritic cell expression of IL-12, which contributed to increased anti-tumor immunity [77]. Another preclinical study demonstrated that blocking CSF-1 limits macrophage infiltration and improves response of mammary carcinomas to chemotherapy (Figure 2B). Targeting TAMs and inflammatory monocytes by inhibiting either CSF1R or CCR2 has been shown to improve chemotherapeutic efficacy, to inhibit metastasis, and to increase antitumor T cell responses [120]. Based on these preclinical studies, several ongoing clinical studies are currently exploring the efficacy of targeting TAMs in cancer patients using anti-CSF1R antibodies in combination with chemotherapy or immunotherapy [121]. For example, a phase I study in patients with solid tumors such as breast and ovarian cancers, evaluated Emactuzumab, an anti-CSF1R treatment, in combination with paclitaxel chemotherapy (Figure 2B). The study demonstrated that this combination reduced the numbers of M2 immunosuppressive macrophages [122]. 

Angiogenic activity is often elevated following chemotherapy, mainly due to increased levels of circulating CEPs or myeloid cells [9,10]. Thus, targeting pro-angiogenic cells in combination with chemotherapy may significantly reduce tumor growth and metastasis, which can further improve therapeutic outcome. For example, in a murine B16 melanoma model, IFN-α combined with dacarbazine treatment was shown to be more effective in reducing tumor size compared to dacarbazine alone (Figure 2C). It was suggested that IFN-α acts through inhibition of G-protein signalling-5 (RGS5), which further reduces pro-angiogenic activity of pericytes and normalizes vasculature [123]. In another study, blocking the mobilization of CEPs in response to chemotherapy using an antiangiogenic drug such as anti-VEGFR2 blocking antibody or anti-VEGF neutralizing antibody resulted in improved therapeutic outcome [124], suggesting that blocking chemotherapy-induced angiogenesis by anti-angiogenic drugs can improve therapy outcome (Figure 2D) [125]. Indeed, several clinical studies using bevacizumab, a neutralizing antibody to VEGF-A, demonstrated improved outcome in combination with chemotherapy probably due to the inhibition of angiogenesis. For example, NSCLC patients treated with bevacizumab combined with platinum-based chemotherapies, such as cisplatin, exhibited increased overall response rate (ORR), with the majority of patients experiencing partial response or stable disease [125]. Likewise, a phase III study of metastatic colorectal cancer patients demonstrated that the use of bevacizumab with napabucasin, an inhibitor of CSCs, and FOLFIRI chemotherapy regimen improved OS (NCT02753127) [126]. In addition, andecaliximab, an anti-MMP-9 antibody along with standard chemotherapies such as oxaliplatin, cisplatin and mFOLFOX6 resulted in reduced angiogenic activity in the TME and further improved outcome in patients with gastric and gastroesophageal junction adenocarcinoma (Figure 2E) [127]. It should be noted that similar to angiogenesis, lymphangiogenesis is also induced in response to paclitaxel chemotherapy. Specifically, paclitaxel induced the secretion of VEGF-C primarily by macrophages. The induction in VEGF-C resulted in increased lymphatic vessels in treated tumors further supporting metastasis. Therefore, blocking VEGF-C-VEGFR3 pathway by anti-VEGFR3 blocking antibodies in combination with paclitaxel chemotherapy significantly reduced metastasis and primary tumor growth in murine lung and breast carcinoma (Figure 2F) [58]. These collective studies demonstrate that blocking host pro-tumorigenic biological pathways induced by chemotherapy can improve outcomes. 

#### 2.4.2. Blunting Host Response with Metronomic Chemotherapy

In the clinic, most chemotherapies are administrated at conventional MTD regimens, to achieve the most effective anti-tumor effect with minimal damage to healthy tissue. However, an alternative drug regimen was introduced two decades ago, namely, metronomic chemotherapy (MC) [128,129]. MC is the administration of chemotherapy at low doses yet in a frequent manner, sometimes on a daily basis, that may reach the same overall dose given by MTD in a given cycle with better tolerance [100,129,130,131,132]. The mechanisms of anti-tumor activity from MC are associated with inhibition of angiogenesis, modulating the immune system and inhibiting CSC enrichment [129,133]. Recent preclinical studies demonstrated that MC is usually associated with better tolerance than MTD, and may avoid the generation of host pro-tumorigenic effects due to the administration of acute doses of the drug [100,134]. For instance, as opposed to MTD gemcitabine which promoted CSC enrichment by inducing stromal ELR+ chemokine paracrine signaling from CAFs, MC gemcitabine prevented CSC enrichment [100]. In another study, MC capecitabine resulted in decreased levels of pro-tumorigenic immune cells in the TME of breast and colon carcinoma compared to the levels of such cells in mice treated with MTD capecitabine [130]. In this study, the authors demonstrated that plasma from the MTD capecitabine-treated mice induced a more tumorigenic and metastatic profile of cancer cells than plasma from mice treated with capecitabine given at a MC regimen. It has been suggested that immunological host effects generated by MTD limit its therapeutic activity, an effect which is overcome by MC [130]. These pro-tumorigenic effects represented by mediators in the plasma of treated mice, were also reported in a pancreatic cancer model treated with gemcitabine administered at MTD compared to MC [135]. Several studies have demonstrated that MC helps to eliminate pro-tumorigenic immune cell types. For example, metronomic doses of paclitaxel have been shown to reduce the frequency of Gr1+ MDSCs in a RET melanoma mouse model [134]. In addition, while cyclophosphamide MTD regimen is usually associated with poor NK cell functions [136], cyclophosphamide administered at a MC regimen has been shown to restore NK cell function and deplete T regulatory cells [137]. Thus, MC inhibits immunosuppressive effects in the tumor and therefore contributes to a better therapeutic outcome. 

In the clinic, a major advantage of MC is its increased tolerability over MTD, thus maintaining a prolonged treatment period along with other treatment modalities. Indeed, several clinical trial protocols using MC alone or in combination with targeted therapies are under evaluation for several cancer indications, some of which have demonstrated encouraging results [138,139,140,141,142,143,144,145,146]. It is possible that the host response generated by the targeted drugs, as previously reported [57], can be blunted by MC, and therefore can improve therapeutic outcome.

#### 2.4.3. Enhancing Chemotherapy-Induced Anti-Tumorigenic Immunological Effects with Immunotherapy 

Immunotherapy has made a huge leap forward in the last decade with the introduction of ICIs in the clinic, resulting in durable complete responses in a subset of patients with advanced metastatic disease for several cancer indications [147]. However, only a limited number of patients respond to ICIs for reasons that are not clear [147]. Moreover, there are clinical reports of tumor hyperprogression, namely, rapid and aggressive progression of tumor growth following treatment, during the course of ICI therapy in some patients [148]. This implies that similar to chemotherapy, immunotherapy may also induce host pro-tumorigenic responses that lead to cancer aggressiveness. Since only a limited number of patients respond to ICI therapy, extensive research efforts are underway to combine ICIs with other drugs in order to increase the proportion of patients who will benefit from this treatment strategy [149]. While it is possible that drug combinations inhibit the unwanted host effects generated in response to ICIs, it is also possible that immunotherapy alters the host immunological responses reported following chemotherapy [57]. Specifically, chemotherapy-induced cytotoxic damage can promote anti-tumor immune activity, due to extensive tumor cell apoptosis and death, resulting in free ATP in the TME. In turn, ATP-polarized CD8+ T cells effectively target tumor cells and promote antitumor activity [150,151]. Therefore, the immune response generated by chemotherapy can be enhanced by ICIs. Several preclinical and clinical studies have demonstrated a synergy for such combination treatment strategies when chemotherapy and/or radiotherapy were used along with ICI [42,152,153,154]. These combination treatments were assessed clinically for several indications including NSCLC and pancreatic cancer, and more clinical studies are ongoing. The use of chemotherapy and ICI combination improved PFS and/or OS and improved clinical outcome when compared to ICI or chemotherapy monotherapy arms. For example, in a prospective clinical study examining the combination of atezolizumab with carboplatin plus nab-paclitaxel chemotherapy, a clinically substantial improvement was demonstrated in both OS and PFS in patients with stage IV non-squamous NSCLC without EGFR and ALK alterations [44]. In another prospective study of patients with previously untreated metastatic squamous NSCLC, the addition of pembrolizumab (anti-PD-1) to chemotherapy of carboplatin plus paclitaxel or nab-paclitaxel resulted in significantly longer OS and PFS than chemotherapy alone [155]. In several retrospective meta-analyses studies examining combination treatments in NSCLC patients, PD-1/PD-L1 inhibitor plus chemotherapy was associated with significantly improved PFS, ORR, and OS in first-line therapy, compared with chemotherapy monotherapy, at the expense of increased treatment-related adverse events [156,157]. In a retrospective clinical study of advanced pancreatic cancer patients treated either with chemotherapy alone or chemotherapy plus ICIs, the combination treatment showed a significantly longer OS and PFS than the chemotherapy monotherapy group, although a similar ORR was found in both arms. These findings suggest that the combination of ICIs with chemotherapy is effective also in advanced pancreatic cancers [158]. Altogether, these and other studies indicate the advantages of combining chemotherapy with ICIs over monotherapy, offering more treatment options that are rationally designed to alter the host immunological response for achieving greater clinical benefit [34,159,160].

## 3. Conclusions and Perspective

Chemotherapy, with all its benefits and disadvantages, is still one of the most common treatment modalities for many cancers. While treatment is usually effective in some patients, there are incidences where therapy ceases to be effective, and patients progress and even sometimes succumb to the disease. Here, we reviewed the literature of possible unique mechanisms to explain tumor re-growth and/or resistance to therapy. While many studies have focused on the effect of the drug on tumor cells, here, we describe studies that specifically focus on the effect of the drug on the host. We explain how such effects contribute to several hallmarks of cancer, such as tumor cell proliferation, angiogenesis, immune modulation and metastasis. Therefore, these studies provide a basis for combining chemotherapy with other treatment modalities to block or blunt the host pro-tumorigenic responses. Targeting host responses that are induced by chemotherapy drugs can lead to a new era for this “old” anti-cancer treatment modality, ultimately increasing clinical benefit for patients. Importantly, while certain chemotherapies may induce common adverse host mediated pro-cancerous effects, it is possible that individual patients react differently to the same chemotherapy drug. Therefore, strategies for screening and deciphering the host response signatures of individual patients are needed. Consequently, host response analysis in the clinical setting may lead to personalized treatments comprising chemotherapy combined with ‘add-on’ drugs tailored for the individual patient [161]. In practice, two prospective clinical studies—CHAMP and PROPHETIC—(NCT04056247) are currently underway [126,162], in which the clinical aspects of host responses to chemotherapy and immunotherapy are being investigated, in order to develop new methodologies and drug combinations for improving treatment outcomes. Such studies and others will pave the way towards better precision medicine in oncology and will lead to the design of clinical trials that test drug combinations based on individual responses to therapy, rather than on empirical strategy.

## References

[1] Maman S., Witz I.P. (2018). A history of exploring cancer in context. Nat. Rev. Cancer.

[2] Gonzalez H., Hagerling C., Werb Z. (2018). Roles of the immune system in cancer: From tumor initiation to metastatic progression. Genes Dev..

[3] Kerbel R.S. (2008). Tumor angiogenesis. N. Engl. J. Med..

[4] Liu T., Zhou L., Li D., Andl T., Zhang Y. (2019). Cancer-Associated Fibroblasts Build and Secure the Tumor Microenvironment. Front. Cell Dev. Biol..

[5] Martin-Padura I., Gregato G., Marighetti P., Mancuso P., Calleri A., Corsini C., Pruneri G., Manzotti M., Lohsiriwat V., Rietjens M. (2012). The white adipose tissue used in lipotransfer procedures is a rich reservoir of CD34+ progenitors able to promote cancer progression. Cancer Res..

[6] Kim J.J., Tannock I.F. (2005). Repopulation of cancer cells during therapy: An important cause of treatment failure. Nat. Rev. Cancer.

[7] Housman G., Byler S., Heerboth S., Lapinska K., Longacre M., Snyder N., Sarkar S. (2014). Drug resistance in cancer: An overview. Cancers.

[8] Shaked Y. (2016). Balancing efficacy of and host immune responses to cancer therapy: The yin and yang effects. Nat. Rev. Clin. Oncol..

[9] Shaked Y., Henke E., Roodhart J.M., Mancuso P., Langenberg M.H., Colleoni M., Daenen L.G., Man S., Xu P., Emmenegger U. (2008). Rapid chemotherapy-induced acute endothelial progenitor cell mobilization: Implications for antiangiogenic drugs as chemosensitizing agents. Cancer Cell.

[10] Shaked Y., Ciarrocchi A., Franco M., Lee C.R., Man S., Cheung A.M., Hicklin D.J., Chaplin D., Foster F.S., Benezra R. (2006). Therapy-induced acute recruitment of circulating endothelial progenitor cells to tumors. Science.

[11] Gingis-Velitski S., Loven D., Benayoun L., Munster M., Bril R., Voloshin T., Alishekevitz D., Bertolini F., Shaked Y. (2011). Host response to short-term, single-agent chemotherapy induces matrix metalloproteinase-9 expression and accelerates metastasis in mice. Cancer Res..

[12] Saggioro M., D’Angelo E., Bisogno G., Agostini M., Pozzobon M. (2020). Carcinoma and Sarcoma Microenvironment at a Glance: Where We Are. Front. Oncol..

[13] Maimela N.R., Liu S., Zhang Y. (2019). Fates of CD8+ T cells in Tumor Microenvironment. Comput. Struct. Biotechnol. J..

[14] Kroemer G., Galluzzi L., Kepp O., Zitvogel L. (2013). Immunogenic cell death in cancer therapy. Annu. Rev. Immunol..

[15] Casares N., Pequignot M.O., Tesniere A., Ghiringhelli F., Roux S., Chaput N., Schmitt E., Hamai A., Hervas-Stubbs S., Obeid M. (2005). Caspase-dependent immunogenicity of doxorubicin-induced tumor cell death. J. Exp. Med..

[16] Eisenbarth S.C. (2019). Dendritic cell subsets in T cell programming: Location dictates function. Nat. Rev. Immunol..

[17] Ma Y., Adjemian S., Mattarollo S.R., Yamazaki T., Aymeric L., Yang H., Portela Catani J.P., Hannani D., Duret H., Steegh K. (2013). Anticancer chemotherapy-induced intratumoral recruitment and differentiation of antigen-presenting cells. Immunity.

[18] Shurin G.V., Tourkova I.L., Kaneno R., Shurin M.R. (2009). Chemotherapeutic agents in noncytotoxic concentrations increase antigen presentation by dendritic cells via an IL-12-dependent mechanism. J. Immunol..

[19] Tanaka H., Matsushima H., Mizumoto N., Takashima A. (2009). Classification of chemotherapeutic agents based on their differential in vitro effects on dendritic cells. Cancer Res..

[20] Chao D., Bahl P., Houlbrook S., Hoy L., Harris A., Austyn J.M. (1999). Human cultured dendritic cells show differential sensitivity to chemotherapy agents as assessed by the MTS assay. Br. J. Cancer.

[21] John J., Ismail M., Riley C., Askham J., Morgan R., Melcher A., Pandha H. (2010). Differential effects of Paclitaxel on dendritic cell function. BMC Immunol..

[22] Zhu Y., Liu N., Xiong S.D., Zheng Y.J., Chu Y.W. (2011). CD4^+^Foxp3^+^ regulatory T-cell impairment by paclitaxel is independent of toll-like receptor 4. Scand. J. Immunol..

[23] Yu Y., Ma X., Gong R., Zhu J., Wei L., Yao J. (2018). Recent advances in CD8(+) regulatory T cell research. Oncol. Lett..

[24] Aldarouish M., Su X., Qiao J., Gao C., Chen Y., Dai A., Zhang T., Shu Y., Wang C. (2019). Immunomodulatory effects of chemotherapy on blood lymphocytes and survival of patients with advanced non-small cell lung cancer. Int. J. Immunopathol. Pharm..

[25] Ruffell B., Au A., Rugo H.S., Esserman L.J., Hwang E.S., Coussens L.M. (2012). Leukocyte composition of human breast cancer. Proc. Natl. Acad. Sci. USA.

[26] Denkert C., von Minckwitz G., Brase J.C., Sinn B.V., Gade S., Kronenwett R., Pfitzner B.M., Salat C., Loi S., Schmitt W.D. (2015). Tumor-infiltrating lymphocytes and response to neoadjuvant chemotherapy with or without carboplatin in human epidermal growth factor receptor 2-positive and triple-negative primary breast cancers. J. Clin. Oncol..

[27] Stockinger B., Omenetti S. (2017). The dichotomous nature of T helper 17 cells. Nat. Rev. Immunol..

[28] Asadzadeh Z., Mohammadi H., Safarzadeh E., Hemmatzadeh M., Mahdian-Shakib A., Jadidi-Niaragh F., Azizi G., Baradaran B. (2017). The paradox of Th17 cell functions in tumor immunity. Cell. Immunol..

[29] Bailey S.R., Nelson M.H., Himes R.A., Li Z., Mehrotra S., Paulos C.M. (2014). Th17 cells in cancer: The ultimate identity crisis. Front. Immunol..

[30] Droeser R.A., Guth U., Eppenberger-Castori S., Stadlmann S., Hirt C., Terracciano L., Singer G. (2013). High IL-17-positive tumor immune cell infiltration is indicative for chemosensitivity of ovarian carcinoma. J. Cancer Res. Clin. Oncol..

[31] Viaud S., Flament C., Zoubir M., Pautier P., LeCesne A., Ribrag V., Soria J.C., Marty V., Vielh P., Robert C. (2011). Cyclophosphamide induces differentiation of Th17 cells in cancer patients. Cancer Res..

[32] Viaud S., Saccheri F., Mignot G., Yamazaki T., Daillere R., Hannani D., Enot D.P., Pfirschke C., Engblom C., Pittet M.J. (2013). The intestinal microbiota modulates the anticancer immune effects of cyclophosphamide. Science.

[33] Lu Y., Zhao Q., Liao J.Y., Song E., Xia Q., Pan J., Li Y., Li J., Zhou B., Ye Y. (2020). Complement Signals Determine Opposite Effects of B Cells in Chemotherapy-Induced Immunity. Cell.

[34] Jiang T., Zhou C., Hu J., Song Y. (2019). Combination immune checkpoint inhibitors with platinum-based chemotherapy in advanced non-small cell lung cancer: What’s known and what’s next. Transl. Lung Cancer Res..

[35] Fregni G., Perier A., Pittari G., Jacobelli S., Sastre X., Gervois N., Allard M., Bercovici N., Avril M.F., Caignard A. (2011). Unique functional status of natural killer cells in metastatic stage IV melanoma patients and its modulation by chemotherapy. Clin. Cancer Res. Off. J. Am. Assoc. Cancer Res..

[36] Vulpis E., Cecere F., Molfetta R., Soriani A., Fionda C., Peruzzi G., Caracciolo G., Palchetti S., Masuelli L., Simonelli L. (2017). Genotoxic stress modulates the release of exosomes from multiple myeloma cells capable of activating NK cell cytokine production: Role of HSP70/TLR2/NF-kB axis. Oncoimmunology.

[37] Luna J.I., Grossenbacher S.K., Sturgill I.R., Ames E., Judge S.J., Bouzid L.A., Darrow M.A., Murphy W.J., Canter R.J. (2019). Bortezomib Augments Natural Killer Cell Targeting of Stem-Like Tumor Cells. Cancers.

[38] McDaniel A.S., Alva A., Zhan T., Xiao H., Cao X., Gursky A., Siddiqui J., Chinnaiyan A.M., Jiang H., Lee C.T. (2016). Expression of PDL1 (B7-H1) Before and After Neoadjuvant Chemotherapy in Urothelial Carcinoma. Eur. Urol. Focus.

[39] Shin J., Chung J.H., Kim S.H., Lee K.S., Suh K.J., Lee J.Y., Kim J.W., Lee J.O., Kim J.W., Kim Y.J. (2019). Effect of Platinum-Based Chemotherapy on PD-L1 Expression on Tumor Cells in Non-small Cell Lung Cancer. Cancer Res. Treat..

[40] Yoneda K., Kuwata T., Kanayama M., Mori M., Kawanami T., Yatera K., Ohguri T., Hisaoka M., Nakayama T., Tanaka F. (2019). Alteration in tumoural PD-L1 expression and stromal CD8-positive tumour-infiltrating lymphocytes after concurrent chemo-radiotherapy for non-small cell lung cancer. Br. J. Cancer.

[41] Lacour M., Hiltbrunner S., Lee S.Y., Soltermann A., Rushing E.J., Soldini D., Weder W., Curioni-Fontecedro A. (2019). Adjuvant Chemotherapy Increases Programmed Death-Ligand 1 (PD-L1) Expression in Non-small Cell Lung Cancer Recurrence. Clin. Lung Cancer.

[42] Timaner M., Kotsofruk R., Raviv Z., Magidey K., Shechter D., Kan T., Nevelsky A., Daniel S., de Vries E.G.E., Zhang T. (2019). Microparticles from tumors exposed to radiation promote immune evasion in part by PD-L1. Oncogene.

[43] Pfirschke C., Engblom C., Rickelt S., Cortez-Retamozo V., Garris C., Pucci F., Yamazaki T., Poirier-Colame V., Newton A., Redouane Y. (2016). Immunogenic Chemotherapy Sensitizes Tumors to Checkpoint Blockade Therapy. Immunity.

[44] West H., McCleod M., Hussein M., Morabito A., Rittmeyer A., Conter H.J., Kopp H.G., Daniel D., McCune S., Mekhail T. (2019). Atezolizumab in combination with carboplatin plus nab-paclitaxel chemotherapy compared with chemotherapy alone as first-line treatment for metastatic non-squamous non-small-cell lung cancer (IMpower130): A multicentre, randomised, open-label, phase 3 trial. Lancet Oncol..

[45] Wang Y., Probin V., Zhou D. (2006). Cancer therapy-induced residual bone marrow injury-Mechanisms of induction and implication for therapy. Curr. Cancer Ther. Rev..

[46] Baras A.S., Drake C., Liu J.J., Gandhi N., Kates M., Hoque M.O., Meeker A., Hahn N., Taube J.M., Schoenberg M.P. (2016). The ratio of CD8 to Treg tumor-infiltrating lymphocytes is associated with response to cisplatin-based neoadjuvant chemotherapy in patients with muscle invasive urothelial carcinoma of the bladder. Oncoimmunology.

[47] Hunder N.N., Wallen H., Cao J., Hendricks D.W., Reilly J.Z., Rodmyre R., Jungbluth A., Gnjatic S., Thompson J.A., Yee C. (2008). Treatment of metastatic melanoma with autologous CD4+ T cells against NY-ESO-1. N. Engl. J. Med..

[48] Matar P., Rozados V.R., Gervasoni S.I., Scharovsky G.O. (2002). Th2/Th1 switch induced by a single low dose of cyclophosphamide in a rat metastatic lymphoma model. Cancer Immunol. Immunother..

[49] Rovati B., Mariucci S., Delfanti S., Grasso D., Tinelli C., Torre C., De Amici M., Pedrazzoli P. (2016). Simultaneous detection of circulating immunological parameters and tumor biomarkers in early stage breast cancer patients during adjuvant chemotherapy. Cell Oncol..

[50] Dong H.P., Elstrand M.B., Holth A., Silins I., Berner A., Trope C.G., Davidson B., Risberg B. (2006). NK- and B-cell infiltration correlates with worse outcome in metastatic ovarian carcinoma. Am. J. Clin. Pathol..

[51] Vigano S., Alatzoglou D., Irving M., Menetrier-Caux C., Caux C., Romero P., Coukos G. (2019). Targeting Adenosine in Cancer Immunotherapy to Enhance T-Cell Function. Front. Immunol..

[52] Mauri C., Menon M. (2015). The expanding family of regulatory B cells. Int. Immunol..

[53] Ziebart A., Huber U., Jeske S., Laban S., Doescher J., Hoffmann T.K., Brunner C., Jackson E.K., Schuler P.J. (2018). The influence of chemotherapy on adenosine-producing B cells in patients with head and neck squamous cell carcinoma. Oncotarget.

[54] Zhang F., Li R., Yang Y., Shi C., Shen Y., Lu C., Chen Y., Zhou W., Lin A., Yu L. (2019). Specific Decrease in B-Cell-Derived Extracellular Vesicles Enhances Post-Chemotherapeutic CD8(+) T Cell Responses. Immunity.

[55] Langers I., Renoux V.M., Thiry M., Delvenne P., Jacobs N. (2012). Natural killer cells: Role in local tumor growth and metastasis. Biologics.

[56] Rey J., Fauriat C., Kochbati E., Orlanducci F., Charbonnier A., D’Incan E., Andre P., Romagne F., Barbarat B., Vey N. (2017). Kinetics of Cytotoxic Lymphocytes Reconstitution after Induction Chemotherapy in Elderly AML Patients Reveals Progressive Recovery of Normal Phenotypic and Functional Features in NK Cells. Front. Immunol..

[57] Shaked Y. (2019). The pro-tumorigenic host response to cancer therapies. Nat. Rev. Cancer.

[58] Alishekevitz D., Gingis-Velitski S., Kaidar-Person O., Gutter-Kapon L., Scherer S.D., Raviv Z., Merquiol E., Ben-Nun Y., Miller V., Rachman-Tzemah C. (2016). Macrophage-Induced Lymphangiogenesis and Metastasis following Paclitaxel Chemotherapy Is Regulated by VEGFR3. Cell Rep..

[59] Shree T., Olson O.C., Elie B.T., Kester J.C., Garfall A.L., Simpson K., Bell-McGuinn K.M., Zabor E.C., Brogi E., Joyce J.A. (2011). Macrophages and cathepsin proteases blunt chemotherapeutic response in breast cancer. Genes Dev..

[60] Akkari L., Gocheva V., Kester J.C., Hunter K.E., Quick M.L., Sevenich L., Wang H.W., Peters C., Tang L.H., Klimstra D.S. (2014). Distinct functions of macrophage-derived and cancer cell-derived cathepsin Z combine to promote tumor malignancy via interactions with the extracellular matrix. Genes Dev..

[61] Karagiannis G.S., Pastoriza J.M., Wang Y., Harney A.S., Entenberg D., Pignatelli J., Sharma V.P., Xue E.A., Cheng E., D’Alfonso T.M. (2017). Neoadjuvant chemotherapy induces breast cancer metastasis through a TMEM-mediated mechanism. Sci. Transl. Med..

[62] Harney A.S., Arwert E.N., Entenberg D., Wang Y., Guo P., Qian B.Z., Oktay M.H., Pollard J.W., Jones J.G., Condeelis J.S. (2015). Real-Time Imaging Reveals Local, Transient Vascular Permeability, and Tumor Cell Intravasation Stimulated by TIE2hi Macrophage-Derived VEGFA. Cancer Discov..

[63] Paulus P., Stanley E.R., Schafer R., Abraham D., Aharinejad S. (2006). Colony-stimulating factor-1 antibody reverses chemoresistance in human MCF-7 breast cancer xenografts. Cancer Res..

[64] DeNardo D.G., Brennan D.J., Rexhepaj E., Ruffell B., Shiao S.L., Madden S.F., Gallagher W.M., Wadhwani N., Keil S.D., Junaid S.A. (2011). Leukocyte complexity predicts breast cancer survival and functionally regulates response to chemotherapy. Cancer Discov..

[65] Zhang Z.J., Bulur P.A., Dogan A., Gastineau D.A., Dietz A.B., Lin Y. (2015). Immune independent crosstalk between lymphoma and myeloid suppressor CD14(+)HLA-DR(low/neg) monocytes mediates chemotherapy resistance. Oncoimmunology.

[66] Bruchard M., Mignot G., Derangere V., Chalmin F., Chevriaux A., Vegran F., Boireau W., Simon B., Ryffel B., Connat J.L. (2013). Chemotherapy-triggered cathepsin B release in myeloid-derived suppressor cells activates the Nlrp3 inflammasome and promotes tumor growth. Nat. Med..

[67] Voloshin T., Alishekevitz D., Kaneti L., Miller V., Isakov E., Kaplanov I., Voronov E., Fremder E., Benhar M., Machluf M. (2015). Blocking IL1beta Pathway Following Paclitaxel Chemotherapy Slightly Inhibits Primary Tumor Growth but Promotes Spontaneous Metastasis. Mol. Cancer Ther..

[68] Dijkgraaf E.M., Heusinkveld M., Tummers B., Vogelpoel L.T., Goedemans R., Jha V., Nortier J.W., Welters M.J., Kroep J.R., van der Burg S.H. (2013). Chemotherapy alters monocyte differentiation to favor generation of cancer-supporting M2 macrophages in the tumor microenvironment. Cancer Res..

[69] Welford A.F., Biziato D., Coffelt S.B., Nucera S., Fisher M., Pucci F., Di Serio C., Naldini L., De Palma M., Tozer G.M. (2011). TIE2-expressing macrophages limit the therapeutic efficacy of the vascular-disrupting agent combretastatin A4 phosphate in mice. J. Clin. Investig..

[70] Mukai K., Tsai M., Saito H., Galli S.J. (2018). Mast cells as sources of cytokines, chemokines, and growth factors. Immunol. Rev..

[71] Liu Z., Zhu Y., Xu L., Zhang J., Xie H., Fu H., Zhou Q., Chang Y., Dai B., Xu J. (2018). Tumor stroma-infiltrating mast cells predict prognosis and adjuvant chemotherapeutic benefits in patients with muscle invasive bladder cancer. Oncoimmunology.

[72] Porcelli L., Iacobazzi R.M., Di Fonte R., Serrati S., Intini A., Solimando A.G., Brunetti O., Calabrese A., Leonetti F., Azzariti A. (2019). CAFs and TGF-beta Signaling Activation by Mast Cells Contribute to Resistance to Gemcitabine/Nabpaclitaxel in Pancreatic Cancer. Cancers.

[73] Bo X., Wang J., Suo T., Ni X., Liu H., Shen S., Li M., Wang Y., Liu H., Xu J. (2018). Tumor-infiltrating mast cells predict prognosis and gemcitabine-based adjuvant chemotherapeutic benefit in biliary tract cancer patients. BMC Cancer.

[74] Wang J.T., Li H., Zhang H., Chen Y.F., Cao Y.F., Li R.C., Lin C., Wei Y.C., Xiang X.N., Fang H.J. (2019). Intratumoral IL17-producing cells infiltration correlate with antitumor immune contexture and improved response to adjuvant chemotherapy in gastric cancer. Ann. Oncol..

[75] Reddy S.M., Reuben A., Barua S., Jiang H., Zhang S., Wang L., Gopalakrishnan V., Hudgens C.W., Tetzlaff M.T., Reuben J.M. (2019). Poor Response to Neoadjuvant Chemotherapy Correlates with Mast Cell Infiltration in Inflammatory Breast Cancer. Cancer Immunol. Res..

[76] Xie H., Li C., Dang Q., Chang L.S., Li L. (2016). Infiltrating mast cells increase prostate cancer chemotherapy and radiotherapy resistances via modulation of p38/p53/p21 and ATM signals. Oncotarget.

[77] Ruffell B., Chang-Strachan D., Chan V., Rosenbusch A., Ho C.M., Pryer N., Daniel D., Hwang E.S., Rugo H.S., Coussens L.M. (2014). Macrophage IL-10 blocks CD8+ T cell-dependent responses to chemotherapy by suppressing IL-12 expression in intratumoral dendritic cells. Cancer Cell.

[78] Chiba S., Baghdadi M., Akiba H., Yoshiyama H., Kinoshita I., Dosaka-Akita H., Fujioka Y., Ohba Y., Gorman J.V., Colgan J.D. (2012). Tumor-infiltrating DCs suppress nucleic acid-mediated innate immune responses through interactions between the receptor TIM-3 and the alarmin HMGB1. Nat. Immunol..

[79] Tesfamariam B. (2016). Involvement of platelets in tumor cell metastasis. Pharmacol. Ther..

[80] Buettner R. (2018). Platelets promoting tumor metastasis: Culprits or victims?. J. Thorac. Dis..

[81] Tian C., Song W., Tian X., Sun Y. (2018). Prognostic significance of platelet-to-lymphocyte ratio in patients with ovarian cancer: A meta-analysis. Eur. J. Clin. Investig..

[82] Wu Y., Li C., Zhao J., Yang L., Liu F., Zheng H., Wang Z., Xu Y. (2016). Neutrophil-to-lymphocyte and platelet-to-lymphocyte ratios predict chemotherapy outcomes and prognosis in patients with colorectal cancer and synchronous liver metastasis. World J. Surg. Oncol..

[83] Wang J., Qu J., Li Z., Che X., Liu J., Teng Y., Jin B., Zhao M., Liu Y., Qu X. (2018). Pretreatment platelet-to-lymphocyte ratio is associated with the response to first-line chemotherapy and survival in patients with metastatic gastric cancer. J. Clin. Lab. Anal..

[84] Vernieri C., Mennitto A., Prisciandaro M., Huber V., Milano M., Rinaldi L., Cona M.S., Maggi C., Ferrari B., Manoukian S. (2018). The neutrophil-to-lymphocyte and platelet-to-lymphocyte ratios predict efficacy of platinum-based chemotherapy in patients with metastatic triple negative breast cancer. Sci. Rep..

[85] Yang Y., Xu H., Zhou L., Deng T., Ning T., Liu R., Zhang L., Wang X., Ge S., Li H. (2018). Platelet to lymphocyte ratio is a predictive marker of prognosis and therapeutic effect of postoperative chemotherapy in non-metastatic esophageal squamous cell carcinoma. Clin. Chim. Acta.

[86] Roxburgh C.S., McMillan D.C. (2014). Cancer and systemic inflammation: Treat the tumour and treat the host. Br. J. Cancer.

[87] Zhou T., Zhan J., Hong S., Hu Z., Fang W., Qin T., Ma Y., Yang Y., He X., Zhao Y. (2015). Ratio of C-Reactive Protein/Albumin is An Inflammatory Prognostic Score for Predicting Overall Survival of Patients with Small-cell Lung Cancer. Sci. Rep..

[88] Nash G.F., Turner L.F., Scully M.F., Kakkar A.K. (2002). Platelets and cancer. Lancet Oncol..

[89] Sylman J.L., Mitrugno A., Tormoen G.W., Wagner T.H., Mallick P., McCarty O.J.T. (2017). Platelet count as a predictor of metastasis and venous thromboembolism in patients with cancer. Converg. Sci. Phys. Oncol..

[90] Paget S. (1989). The distribution of secondary growths in cancer of the breast. Cancer Metastasis Rev..

[91] Kaplan R.N., Riba R.D., Zacharoulis S., Bramley A.H., Vincent L., Costa C., MacDonald D.D., Jin D.K., Shido K., Kerns S.A. (2005). VEGFR1-positive haematopoietic bone marrow progenitors initiate the pre-metastatic niche. Nature.

[92] Daenen L.G., Roodhart J.M., van Amersfoort M., Dehnad M., Roessingh W., Ulfman L.H., Derksen P.W., Voest E.E. (2011). Chemotherapy enhances metastasis formation via VEGFR-1-expressing endothelial cells. Cancer Res..

[93] Zenitani M., Nojiri T., Hosoda H., Kimura T., Uehara S., Miyazato M., Okuyama H., Kangawa K. (2018). Chemotherapy can promote liver metastasis by enhancing metastatic niche formation in mice. J. Surg. Res..

[94] Timaner M., Bril R., Kaidar-Person O., Rachman-Tzemah C., Alishekevitz D., Kotsofruk R., Miller V., Nevelsky A., Daniel S., Raviv Z. (2015). Dequalinium blocks macrophage-induced metastasis following local radiation. Oncotarget.

[95] Keklikoglou I., Cianciaruso C., Guc E., Squadrito M.L., Spring L.M., Tazzyman S., Lambein L., Poissonnier A., Ferraro G.B., Baer C. (2019). Chemotherapy elicits pro-metastatic extracellular vesicles in breast cancer models. Nat. Cell Biol..

[96] Chan T.S., Shaked Y., Tsai K.K. (2019). Targeting the Interplay between Cancer Fibroblasts, Mesenchymal Stem Cells, and Cancer Stem Cells in Desmoplastic Cancers. Front. Oncol..

[97] Shibue T., Weinberg R.A. (2017). EMT, CSCs, and drug resistance: The mechanistic link and clinical implications. Nat. Rev. Clin. Oncol..

[98] Skolekova S., Matuskova M., Bohac M., Toro L., Durinikova E., Tyciakova S., Demkova L., Gursky J., Kucerova L. (2016). Cisplatin-induced mesenchymal stromal cells-mediated mechanism contributing to decreased antitumor effect in breast cancer cells. Cell Commun. Signal..

[99] Timaner M., Letko-Khait N., Kotsofruk R., Benguigui M., Beyar-Katz O., Rachman-Tzemah C., Raviv Z., Bronshtein T., Machluf M., Shaked Y. (2018). Therapy-Educated Mesenchymal Stem Cells Enrich for Tumor-Initiating Cells. Cancer Res..

[100] Chan T.S., Hsu C.C., Pai V.C., Liao W.Y., Huang S.S., Tan K.T., Yen C.J., Hsu S.C., Chen W.Y., Shan Y.S. (2016). Metronomic chemotherapy prevents therapy-induced stromal activation and induction of tumor-initiating cells. J. Exp. Med..

[101] Su S., Chen J., Yao H., Liu J., Yu S., Lao L., Wang M., Luo M., Xing Y., Chen F. (2018). CD10^+^ GPR77^+^ Cancer-Associated Fibroblasts Promote Cancer Formation and Chemoresistance by Sustaining Cancer Stemness. Cell.

[102] Roodhart J.M., Daenen L.G., Stigter E.C., Prins H.J., Gerrits J., Houthuijzen J.M., Gerritsen M.G., Schipper H.S., Backer M.J., van Amersfoort M. (2011). Mesenchymal stem cells induce resistance to chemotherapy through the release of platinum-induced fatty acids. Cancer Cell.

[103] Lis R., Touboul C., Mirshahi P., Ali F., Mathew S., Nolan D.J., Maleki M., Abdalla S.A., Raynaud C.M., Querleu D. (2011). Tumor associated mesenchymal stem cells protects ovarian cancer cells from hyperthermia through CXCL12. Int. J. Cancer.

[104] Scherzed A., Hackenberg S., Froelich K., Kessler M., Koehler C., Hagen R., Radeloff A., Friehs G., Kleinsasser N. (2011). BMSC enhance the survival of paclitaxel treated squamous cell carcinoma cells in vitro. Cancer Biol. Ther..

[105] Zeng J., Chen S., Li C., Ye Z., Lin B., Liang Y., Wang B., Ma Y., Chai X., Zhang X. (2020). Mesenchymal stem/stromal cells-derived IL-6 promotes nasopharyngeal carcinoma growth and resistance to cisplatin via upregulating CD73 expression. J. Cancer.

[106] Tu B., Zhu J., Liu S., Wang L., Fan Q., Hao Y., Fan C., Tang T.T. (2016). Mesenchymal stem cells promote osteosarcoma cell survival and drug resistance through activation of STAT3. Oncotarget.

[107] Cao Y. (2019). Adipocyte and lipid metabolism in cancer drug resistance. J. Clin. Investig..

[108] Lehuede C., Li X., Dauvillier S., Vaysse C., Franchet C., Clement E., Esteve D., Longue M., Chaltiel L., Le Gonidec S. (2019). Adipocytes promote breast cancer resistance to chemotherapy, a process amplified by obesity: Role of the major vault protein (MVP). Breast Cancer Res..

[109] Okumura T., Ohuchida K., Sada M., Abe T., Endo S., Koikawa K., Iwamoto C., Miura D., Mizuuchi Y., Moriyama T. (2017). Extra-pancreatic invasion induces lipolytic and fibrotic changes in the adipose microenvironment, with released fatty acids enhancing the invasiveness of pancreatic cancer cells. Oncotarget.

[110] De Angel R.E., Blando J.M., Hogan M.G., Sandoval M.A., Lansakara P.D., Dunlap S.M., Hursting S.D., Cui Z. (2013). Stearoyl gemcitabine nanoparticles overcome obesity-induced cancer cell resistance to gemcitabine in a mouse postmenopausal breast cancer model. Cancer Biol. Ther..

[111] Chi M., Chen J., Ye Y., Tseng H.Y., Lai F., Tay K.H., Jin L., Guo S.T., Jiang C.C., Zhang X.D. (2014). Adipocytes contribute to resistance of human melanoma cells to chemotherapy and targeted therapy. Curr. Med. Chem..

[112] Yang J., Zaman M.M., Vlasakov I., Roy R., Huang L., Martin C.R., Freedman S.D., Serhan C.N., Moses M.A. (2019). Adipocytes promote ovarian cancer chemoresistance. Sci. Rep..

[113] Harbuzariu A., Gonzalez-Perez R.R. (2018). Leptin-Notch axis impairs 5-fluorouracil effects on pancreatic cancer. Oncotarget.

[114] Bartucci M., Svensson S., Ricci-Vitiani L., Dattilo R., Biffoni M., Signore M., Ferla R., De Maria R., Surmacz E. (2010). Obesity hormone leptin induces growth and interferes with the cytotoxic effects of 5-fluorouracil in colorectal tumor stem cells. Endocr. Relat. Cancer.

[115] Liu Z., Xu J., He J., Liu H., Lin P., Wan X., Navone N.M., Tong Q., Kwak L.W., Orlowski R.Z. (2015). Mature adipocytes in bone marrow protect myeloma cells against chemotherapy through autophagy activation. Oncotarget.

[116] Roodhart J.M., Langenberg M.H., Vermaat J.S., Lolkema M.P., Baars A., Giles R.H., Witteveen E.O., Voest E.E. (2010). Late release of circulating endothelial cells and endothelial progenitor cells after chemotherapy predicts response and survival in cancer patients. Neoplasia.

[117] Calleri A., Bono A., Bagnardi V., Quarna J., Mancuso P., Rabascio C., Dellapasqua S., Campagnoli E., Shaked Y., Goldhirsch A. (2009). Predictive Potential of Angiogenic Growth Factors and Circulating Endothelial Cells in Breast Cancer Patients Receiving Metronomic Chemotherapy Plus Bevacizumab. Clin. Cancer Res. Off. J. Am. Assoc. Cancer Res..

[118] Voloshin T., Gingis-Velitski S., Bril R., Benayoun L., Munster M., Milsom C., Man S., Kerbel R.S., Shaked Y. (2011). G-CSF supplementation with chemotherapy can promote revascularization and subsequent tumor regrowth: Prevention by a CXCR4 antagonist. Blood.

[119] Shaked Y., Tang T., Woloszynek J., Daenen L.G., Man S., Xu P., Cai S.R., Arbeit J.M., Voest E.E., Chaplin D.J. (2009). Contribution of granulocyte colony-stimulating factor to the acute mobilization of endothelial precursor cells by vascular disrupting agents. Cancer Res..

[120] Mitchem J.B., Brennan D.J., Knolhoff B.L., Belt B.A., Zhu Y., Sanford D.E., Belaygorod L., Carpenter D., Collins L., Piwnica-Worms D. (2013). Targeting tumor-infiltrating macrophages decreases tumor-initiating cells, relieves immunosuppression, and improves chemotherapeutic responses. Cancer Res..

[121] Anfray C., Ummarino A., Andon F.T., Allavena P. (2019). Current Strategies to Target Tumor-Associated-Macrophages to Improve Anti-Tumor Immune Responses. Cells.

[122] Gomez-Roca C.A., Italiano A., Le Tourneau C., Cassier P.A., Toulmonde M., D’Angelo S.P., Campone M., Weber K.L., Loirat D., Cannarile M.A. (2019). Phase I Study of Emactuzumab Single Agent or in Combination with Paclitaxel in Patients with Advanced/Metastatic Solid Tumors Reveals Depletion of Immunosuppressive M2-like Macrophages. Ann. Oncol..

[123] Liu P., Zhang C., Chen J., Zhang R., Ren J., Huang Y., Zhu F., Li Z., Wu G. (2011). Combinational therapy of interferon-alpha and chemotherapy normalizes tumor vasculature by regulating pericytes including the novel marker RGS5 in melanoma. J. Immunother..

[124] Zhao X., Liu H.Q., Li J., Liu X.L. (2016). Endothelial progenitor cells promote tumor growth and progression by enhancing new vessel formation. Oncol. Lett..

[125] Rajabi M., Mousa S.A. (2017). The Role of Angiogenesis in Cancer Treatment. Biomedicines.

[126] ClinicalTrials.gov. https://clinicaltrials.gov/.

[127] Shah M.A., Starodub A., Sharma S., Berlin J., Patel M., Wainberg Z.A., Chaves J., Gordon M., Windsor K., Brachmann C.B. (2018). Andecaliximab/GS-5745 Alone and Combined with mFOLFOX6 in Advanced Gastric and Gastroesophageal Junction Adenocarcinoma: Results from a Phase I Study. Clin. Cancer Res. Off. J. Am. Assoc. Cancer Res..

[128] Benzekry S., Pasquier E., Barbolosi D., Lacarelle B., Barlesi F., Andre N., Ciccolini J. (2015). Metronomic reloaded: Theoretical models bringing chemotherapy into the era of precision medicine. Semin. Cancer Biol..

[129] Simsek C., Esin E., Yalcin S. (2019). Metronomic Chemotherapy: A Systematic Review of the Literature and Clinical Experience. J. Oncol..

[130] Shaked Y., Pham E., Hariharan S., Magidey K., Beyar-Katz O., Xu P., Man S., Wu F.T., Miller V., Andrews D. (2016). Evidence Implicating Immunological Host Effects in the Efficacy of Metronomic Low-Dose Chemotherapy. Cancer Res..

[131] Chen C.S., Doloff J.C., Waxman D.J. (2014). Intermittent metronomic drug schedule is essential for activating antitumor innate immunity and tumor xenograft regression. Neoplasia.

[132] Tongu M., Harashima N., Monma H., Inao T., Yamada T., Kawauchi H., Harada M. (2013). Metronomic chemotherapy with low-dose cyclophosphamide plus gemcitabine can induce anti-tumor T cell immunity in vivo. Cancer Immunol. Immunother..

[133] Biziota E., Mavroeidis L., Hatzimichael E., Pappas P. (2017). Metronomic chemotherapy: A potent macerator of cancer by inducing angiogenesis suppression and antitumor immune activation. Cancer Lett..

[134] Umansky V., Sevko A. (2012). Overcoming immunosuppression in the melanoma microenvironment induced by chronic inflammation. Cancer Immunol. Immunother..

[135] Hasnis E., Alishekevitz D., Gingis-Veltski S., Bril R., Fremder E., Voloshin T., Raviv Z., Karban A., Shaked Y. (2014). Anti-Bv8 antibody and metronomic gemcitabine improve pancreatic adenocarcinoma treatment outcome following weekly gemcitabine therapy. Neoplasia.

[136] Kareva I., Waxman D.J., Lakka Klement G. (2015). Metronomic chemotherapy: An attractive alternative to maximum tolerated dose therapy that can activate anti-tumor immunity and minimize therapeutic resistance. Cancer Lett..

[137] Ghiringhelli F., Menard C., Puig P.E., Ladoire S., Roux S., Martin F., Solary E., Le Cesne A., Zitvogel L., Chauffert B. (2007). Metronomic cyclophosphamide regimen selectively depletes CD4+CD25+ regulatory T cells and restores T and NK effector functions in end stage cancer patients. Cancer Immunol. Immunother..

[138] Krajnak S., Schnatz C., Almstedt K., Brenner W., Haertner F., Heimes A.S., Lebrecht A., Makris G.M., Schwab R., Hasenburg A. (2020). Low-dose metronomic chemotherapy as an efficient treatment option in metastatic breast cancer-results of an exploratory case-control study. Breast Cancer Res. Treat..

[139] Orlando L., Lorusso V., Giotta F., Di Maio M., Schiavone P., Fedele P., Quaranta A., Caliolo C., Ciccarese M., Cinefra M. (2020). Metronomic oral chemotherapy with cyclophosphamide plus capecitabine combined with trastuzumab (HEX) as first line therapy of HER-2 positive advanced breast cancer: A phase II trial of the Gruppo Oncologico Italia Meridionale (GOIM). Breast.

[140] Patil V.M., Noronha V., Joshi A., Dhumal S., Mahimkar M., Bhattacharjee A., Gota V., Pandey M., Menon N., Mahajan A. (2019). Phase I/II Study of Palliative Triple Metronomic Chemotherapy in Platinum-Refractory/Early-Failure Oral Cancer. J. Clin. Oncol..

[141] Cremolini C., Marmorino F., Bergamo F., Aprile G., Salvatore L., Masi G., Dell’Aquila E., Antoniotti C., Murgioni S., Allegrini G. (2019). Phase II randomised study of maintenance treatment with bevacizumab or bevacizumab plus metronomic chemotherapy after first-line induction with FOLFOXIRI plus Bevacizumab for metastatic colorectal cancer patients: The MOMA trial. Eur. J. Cancer.

[142] Wildiers H., Tryfonidis K., Dal Lago L., Vuylsteke P., Curigliano G., Waters S., Brouwers B., Altintas S., Touati N., Cardoso F. (2018). Pertuzumab and trastuzumab with or without metronomic chemotherapy for older patients with HER2-positive metastatic breast cancer (EORTC 75111-10114): An open-label, randomised, phase 2 trial from the Elderly Task Force/Breast Cancer Group. Lancet Oncol..

[143] Montagna E., Bagnardi V., Cancello G., Sangalli C., Pagan E., Iorfida M., Mazza M., Mazzarol G., Dellapasqua S., Munzone E. (2018). Metronomic Chemotherapy for First-Line Treatment of Metastatic Triple-Negative Breast Cancer: A Phase II Trial. Breast Care.

[144] Launay S., Sabatier R., Brunelle S., Esterni B., Tarpin C., Viret F., Gravis G., Cappiello M., Provansal M., Extra J.M. (2016). METRO1: A Phase I Study of Metronomic Chemotherapy in Adults with Advanced Refractory Solid Tumors. Anticancer Res..

[145] Elharrar X., Barbolosi D., Ciccolini J., Meille C., Faivre C., Lacarelle B., Andre N., Barlesi F. (2016). A phase Ia/Ib clinical trial of metronomic chemotherapy based on a mathematical model of oral vinorelbine in metastatic non-small cell lung cancer and malignant pleural mesothelioma: Rationale and study protocol. BMC Cancer.

[146] Kerbel R.S., Grothey A. (2015). Gastrointestinal cancer: Rationale for metronomic chemotherapy in phase III trials. Nat. Rev. Clin. Oncol..

[147] Esfahani K., Roudaia L., Buhlaiga N., Del Rincon S.V., Papneja N., Miller W.H. (2020). A review of cancer immunotherapy: From the past, to the present, to the future. Curr. Oncol..

[148] Fuentes-Antras J., Provencio M., Diaz-Rubio E. (2018). Hyperprogression as a distinct outcome after immunotherapy. Cancer Treat. Rev..

[149] Sharma P., Allison J.P. (2015). Immune checkpoint targeting in cancer therapy: Toward combination strategies with curative potential. Cell.

[150] Ma Y., Yamazaki T., Yang H., Kepp O., Galluzzi L., Zitvogel L., Smyth M.J., Kroemer G. (2013). Tumor necrosis factor is dispensable for the success of immunogenic anticancer chemotherapy. Oncoimmunology.

[151] Aymeric L., Apetoh L., Ghiringhelli F., Tesniere A., Martins I., Kroemer G., Smyth M.J., Zitvogel L. (2010). Tumor cell death and ATP release prime dendritic cells and efficient anticancer immunity. Cancer Res..

[152] Heinhuis K.M., Ros W., Kok M., Steeghs N., Beijnen J.H., Schellens J.H.M. (2019). Enhancing antitumor response by combining immune checkpoint inhibitors with chemotherapy in solid tumors. Ann. Oncol..

[153] Yan Y., Kumar A.B., Finnes H., Markovic S.N., Park S., Dronca R.S., Dong H. (2018). Combining Immune Checkpoint Inhibitors with Conventional Cancer Therapy. Front. Immunol..

[154] Kasmann L., Eze C., Dantes M., Roengvoraphoj O., Niyazi M., Belka C., Manapov F. (2019). State of clinical research of radiotherapy/chemoradiotherapy and immune checkpoint inhibitor therapy combinations in solid tumours-a German radiation oncology survey. Eur. J. Cancer.

[155] Paz-Ares L., Luft A., Vicente D., Tafreshi A., Gumus M., Mazieres J., Hermes B., Cay Senler F., Csoszi T., Fulop A. (2018). Pembrolizumab plus Chemotherapy for Squamous Non-Small-Cell Lung Cancer. N. Engl. J. Med..

[156] Zhou Y., Chen C., Zhang X., Fu S., Xue C., Ma Y., Fang W., Yang Y., Hou X., Huang Y. (2018). Immune-checkpoint inhibitor plus chemotherapy versus conventional chemotherapy for first-line treatment in advanced non-small cell lung carcinoma: A systematic review and meta-analysis. J. Immunother. Cancer.

[157] Addeo A., Banna G.L., Metro G., Di Maio M. (2019). Chemotherapy in Combination with Immune Checkpoint Inhibitors for the First-Line Treatment of Patients with Advanced Non-small Cell Lung Cancer: A Systematic Review and Literature-Based Meta-Analysis. Front. Oncol..

[158] Ma J., Sun D., Wang J., Han C., Qian Y., Chen G., Li X., Zhang J., Cui P., Du W. (2020). Immune checkpoint inhibitors combined with chemotherapy for the treatment of advanced pancreatic cancer patients. Cancer Immunol. Immunother..

[159] Gridelli C., Casaluce F. (2018). The combination strategies will be ready the right first-line choice for squamous lung cancer patients?. Transl. Lung Cancer Res..

[160] Gravara L.D., Battiloro C., Cantile R., Letizia A., Vitiello F., Montesarchio V., Rocco D. (2020). Chemotherapy and/or immune checkpoint inhibitors in NSCLC first-line setting: What is the best approach?. Lung Cancer Manag..

[161] Andre N., Carre M., Pasquier E. (2014). Metronomics: Towards personalized chemotherapy?. Nat. Rev. Clin. Oncol..

[162] Hau S.O., Petersson A., Nodin B., Karnevi E., Boman K., Williamsson C., Eberhard J., Leandersson K., Gisselsson D., Heby M. (2020). Chemotherapy, host response and molecular dynamics in periampullary cancer: The CHAMP study. BMC Cancer.

